# Degradation and Detection of Endocrine Disruptors by Laccase-Mimetic Polyoxometalates

**DOI:** 10.3389/fchem.2022.854045

**Published:** 2022-02-16

**Authors:** Kun Chen, Shengqiu Liu, Qiongyu Zhang

**Affiliations:** ^1^ South China Advanced Institute for Soft Matter Science and Technology, School of Molecular Science and Engineering, South China University of Technology, Guangzhou, China; ^2^ State Key Laboratory of Luminescent Materials and Devices and Guangdong Provincial Key Laboratory of Functional and Intelligent Hybrid Materials and Devices, South China University of Technology, Guangzhou, China

**Keywords:** endocrine disruptors, polyoxometalates (POMs), water contaminants, laccase-mimetic degradation, nanozymes

## Abstract

Endocrine disruptors are newly identified water contaminants and immediately caught worldwide concern. An effort has been made to degrade endocrine disruptors in the water body by relying on laccase-assisted approaches, including laccase-mediated catalytic systems, immobilized laccase catalytic systems, and nano-catalytic systems based on atypical protein enzymes. Analogous to laccases, polyoxometalates (POMs) have a similar size as these enzymes. They are also capable of using oxygen as an electron acceptor, which could assist the removal of endocrine disruptors in water. This perspective begins with a brief introduction to endocrine disruptors and laccases, summarizes current approaches employing laccases, and focuses on the nano-catalytic systems that mimic the function of laccases. Among the inorganic nanoparticles, POMs meet the design requirements and are easy for large-scale production. The catalytic performance of POMs in water treatment is highlighted, and an example of using polyoxovanadates for endocrine disruptor degradation is given at the end of this perspective. Exploring laccase-mimetic POMs will give key insights into the degradation of emergent water contaminants.

## Introduction

**FIGURE 1:**
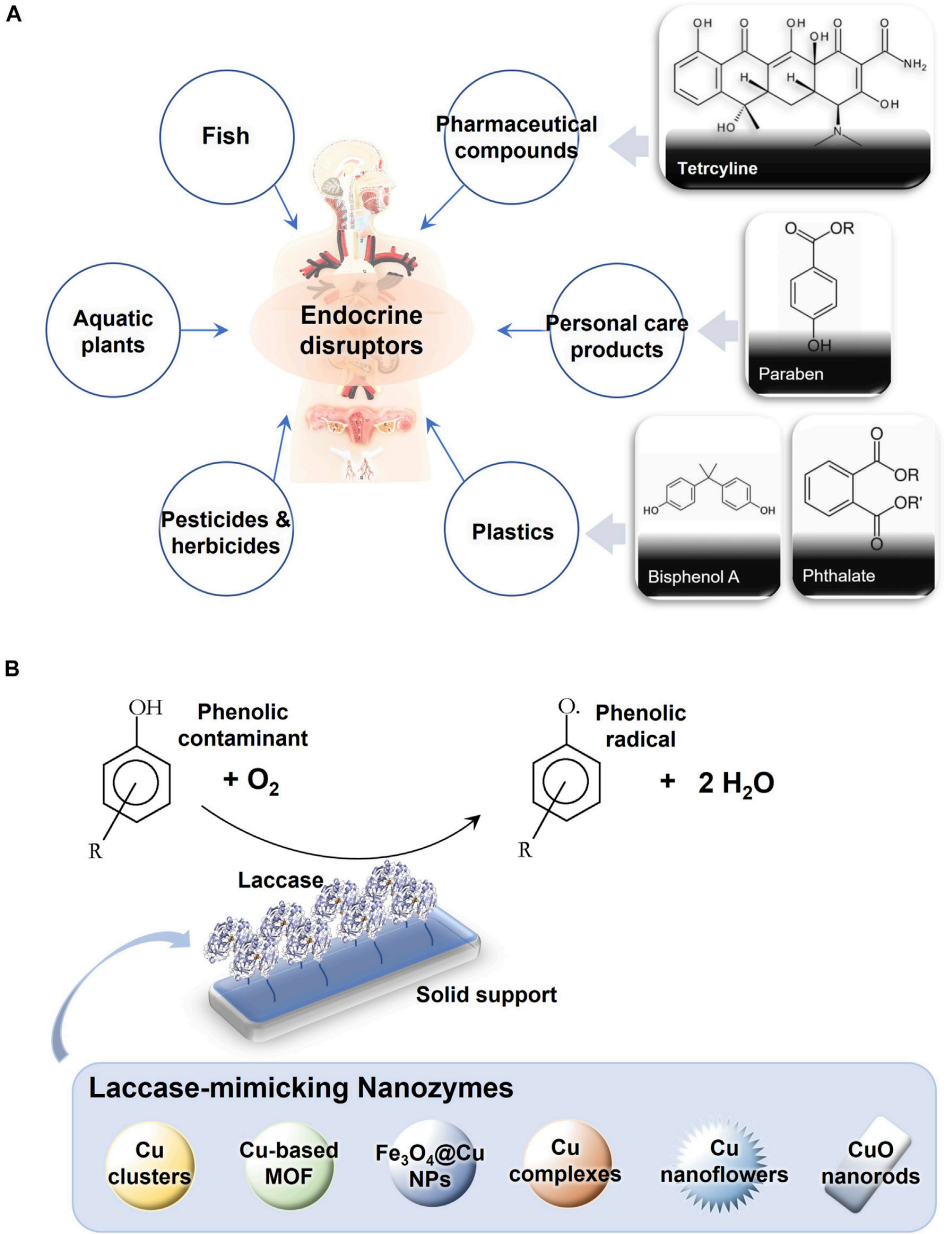
**(A)** Common sources and typical structures of endocrine disruptors in water environment. **(B)** Scheme of immobilized laccase-assisted degradation of phenolic contaminants and the representative nanozymes that have been reported the catalytic activities of mimicking laccase.

Endocrine disruptors are emergent water contaminants which frequently found in every aspect of human life, including some plastic bottles and containers, fungicides, disinfectants, anti-viral agents, pharmaceutical drugs for oral analgesic and mild anesthetic, and cosmetics and skin care products (Figure 1A) (Bilal et al., 2019a). Some of the endocrine disruptors are phenol products that are analogous to natural hormones due to their capability of acting like natural hormones and disrupting the endocrine system (Barrios-Estrada et al., 2018). Endocrine disruptors receive more attention than other phenols because of their appearance in various sources and unique interferences with natural hormones in interaction with corresponding receptors that result in an altered cellular signal and subsequent a failure in the body. The persistence of endocrine disruptors in water bodies has raised particular concern because of the widespread and continuing sewage discharge.

Enzymatic degradation approaches have been developed for addressing this growing issue (Bilal et al., 2019b). Laccase catalyzed reaction effectively removes many kinds of trace pollutants, which are difficult to degrade in wastewater, especially some phenolic endocrine disruptors (Mate and Alcalde, 2017; Janusz et al., 2020). Laccases catalyze one-electron oxidation of a broad range of these phenol substrates and release water as the by-product (Figure 1B). As an eco-friendly and versatile biocatalyst, laccases have been applied in enzymatic bioremediation of the water bodies (Sharma et al., 2018; Morsi et al., 2020). To extend the using range in the harsh environment, a set of nanosystems, mostly nanozymes, are designed to mimic the function of laccases in wastewater treatment (Zhou et al., 2017). Polyoxometalates (POMs) are nanosized soluble molecular metal-oxo clusters with well-defined structures. Each metal-oxo cluster comprises an array of corner-sharing and edge-sharing pseudo-octahedral MO_6_ (M = Mo, W, V, Nb, etc.) units (Gumerova and Rompel, 2018). POMs have demonstrated promising catalytic activities over a wide range of catalysis fields (Anyushin et al., 2018). POMs for constructing the nanocatalytic system can be directly used to catalyze the same substrates as laccases. Meanwhile, POMs are much more cost-effective for production and robust for working at high temperature, high pressure, and extreme pH conditions (Liu et al., 2020). In this perspective, the current development in this field is briefly summarized, and the scientific and technological challenges are outlined. Exploring laccase-mimetic POMs would spark future research interest in advancing the technic for the removal of emergent water contaminants.

### Endocrine Disruptors in Water

Environmental endocrine disruptors, a type of pollutant in water, are a classification of exotic chemicals that alter or interact with the endocrine systems of vertebrates and invertebrates (Figure 1A). Endocrine disruptors belong to the endocrine organic chemicals, but differ from natural phytoestrogens in that they mimic, block, or alter the actions of normal hormones (Landrigan et al., 2018). Endocrine disruptors have shown the potential to interact with biological systems, such as the hormonal system and nervous system, and cause various complications, such as neurodevelopmental toxicity and Parkinson’s disease (Barrios-Estrada et al., 2018). Endocrine disruptors have a variety of mechanisms of action. One pathway is through the direct interaction with a given estrogen receptor, which may interfere with or regulate downstream gene expression. For example, most endocrine disruptor-related reproductive and developmental defects are thought to be due to endocrine disruptors interfering with the function of estrogen receptors and androgen receptors, thereby interfering with the regular activity of estrogen and androgen ligands (Barrios-Estrada et al., 2018). In addition to sex steroid receptors, the estrogen receptor superfamily includes transcription factors that play a key role in integrating complex metabolic homeostasis and development. The ability of endocrine disruptors to interact with these estrogen receptors is supported and explained by metabolic disorders in experimental and epidemiological studies (Barrios-Estrada et al., 2018). During early development, the exposure of even extremely low doses of endocrine disruptors will likely lead to permanent impairments in fetus organ function and increase their disease risk. In addition, many endocrine disruptors are also developmental neurotoxicants and can be stored in animal and human fats for years (Schug et al., 2011). These chemicals include bisphenol A, phthalates, and dioxins.

Water pollution has become a pressing issue as populations grow and industrial production expands. This growing problem is often linked to poor wastewater management, outdated infrastructure, factories, and limited treatment strategies. Most endocrine disruptors come from products used to combat unfavorable wildlife and agricultural threats, such as pesticides and fungicides, as well as various synthetic products used in the plastics industry (bisphenol A or phthalates), insulation materials (polychlorinated biphenyls), and brominated flame retardants (Bilal et al., 2019a). These chemicals are manufactured in the high output of millions of kilograms per year and have caused substantial impacts on our daily life. They are everywhere around people, in consumer products such as perfumes, shampoos, soaps, plastics, and food containers. Another problem associated with such chemicals is that they degrade very slowly or are not photodegradable (Bilal et al., 2019b). Therefore, endocrine disruptors have become a significant public health problem globally due to their high stability, low degradation, high toxicity, and persistence in the environment. Biological technics for the degradation of these pollutants using oxidoreductases are a promising area of research (Cabana et al., 2007a; Cabana et al., 2007b; Torres-Duarte et al., 2012).

### Laccase-Assisted Detection and Degradation

Laccase is a group of enzymes with a wide taxonomic distribution that belongs to the copper oxidases (MCOs) superfamily (Mate and Alcalde, 2017; Janusz et al., 2020). MCOs reduce oxygen molecules to water without releasing harmful substances, including those species often generated during oxygen reduction (Figure 1B), such as the partially reduced products of O_2_, reactive oxygen species (ROS). Laccases are widely distributed in nature. Higher plants, bacteria, lichens, sponges, and fungi, especially white rot fungi, can produce laccases with different biological functions and substrate diversity (Mate and Alcalde, 2017). Aromatic compounds (e.g., catechol and hydroquinone, methoxy substituted phenols, diamines, and phenylthiols), organometals ([Fe(EDTA)]^2−^ and [W(CN)_8_]^4−^), and metal ions are all the substrates of laccases.

The remarkable broad substrate specificity of laccase aroused the attention of those who are worried about the environment. Over the past few decades, laccases have been used as a biocatalyst to detect and reduce pollutants by removing electrons from these organic substrates and blocking the entry of these contaminants into the water bodies. The laccase-involving enzymolysis approach has been used in different industrial applications to replace traditional chemical processes in the paper, textile, cosmetics, paint, pulp, furniture, and pharmaceutical factories (Sharma et al., 2018; Morsi et al., 2020). To develop a robust laccase-based biocatalytic platform, the enzymes are normally immobilized on a support matrix to address the limitations related to enzyme reusability and recycling (Lassouane et al., 2019; Zhou et al., 2021). The stability and resistance of laccase (e.g., isolated from the basidiomycete *Trametes versicolor*) to protease is increased by the immobilization on a solid carrier (Sharma et al., 2018). Several methods for immobilizing enzymes have been developed, such as covalently attaching to solid carriers, solid carrier adsorption methods, embedding in polymeric gels, cross-linking with biofunctional reagents, and embedding in solid carriers (Bilal et al., 2017; Shakerian et al., 2020). Laccase immobilization is a promising water purification technology. Compared with free laccase, the reusability of immobilized laccase makes it more advantageous in the practical application of water purification (Lassouane et al., 2019; Lou et al., 2020; Masjoudi et al., 2021).

In recent years, nanocarriers have been engineered to immobilize and support enzymes, which greatly advanced traditional enzyme-immobilization methods. Many enzyme systems based on nanostructures are designed and used to detect a variety of organic pollutants and degrade them efficiently into harmless smaller intermediates (Alarcón-Payán et al., 2017; Chen C. et al., 2018; Masjoudi et al., 2021; Qiu et al., 2021). Furthermore, the attachment of laccase to its nanocarrier not only reduces its mobilization, but also increases activity and stability of the enzyme (Koyani and Vazquez-Duhalt, 2016; Costa et al., 2019). It was reported that the electrode modified with laccase-immobilizing polyaniline/magnetic graphene exhibited superior electrical properties, high detection sensitivity to hydroquinone, low detection limit, and wide linear range (Lou et al., 2020). Many carrier nanomaterials for laccase immobilization have been engineered. Co-immobilization of laccase and 2,2-binamine-di-3-ethylbenzothiazolin-6-sulfonic acid (ABTS) onto amino-functionalized ionic liquid-modified magnetic chitosan nanoparticles improves the capability of biocatalyst for the pollutant removal of bisphenol A, indole, and anthracene (Qiu et al., 2021). Functionalized multi-walled carbon nanotubes (CNTs) are used as nanocarriers for laccase immobilization to enhance the biocatalytic sustainability of laccase (Costa et al., 2019). In another work, laccase was also cross-linked onto hollow mesoporous carbon spheres (HMCs) for antibiotic degradation and removal from the aqueous phase (Shao et al., 2019). A biomimetic dynamic membrane (BDM) fabricated by using carbon nanotubes (CNTs) and laccases (Chen et al., 2019; Bilal et al., 2021), was proved to be very effective for wastewater treatment (Shao et al., 2019). Quite a few reviews have summarized the progress in nanoengineered laccases-advanced biotechnology (Bilal et al., 2021).

The support nanomaterials used for enzyme-immobilization are expected to be low cost and have a large enough surface area to avoid diffusion limitations of substrates and products of enzyme reaction. Meanwhile, the catalytic efficiency of the enzyme is anticipated to be improved by the immobilization to a solid surface. In general, enzymes are immobilized in various ways: binding to affinity labels, adsorption on substrates, and covalently anchoring to carriers (Shakerian et al., 2020). The immobilization model of laccase with its carriers greatly impacts on the properties of the enzyme (Bilal et al., 2017). At least, immobilization should not affect the conformation and activity of the enzyme, and the activity of immobilized enzymes should be retained for a longer time than that of free enzymes. The degradation of foreign biological compounds using immobilized enzymes may prove economical because of their enhanced stability and reusability. However, the immobilized enzyme cannot be recycled and reused too many times. Sometimes, intracellular enzymes do not work well in cell-free systems. Therefore, a group of nanomaterials was engineered to mimic the function of protein enzymes (Chen et al., 2019; Bilal et al., 2021), a relatively new strategy we are going to discuss in the next part.

### Laccase-Mimicking Nanozymes

Nanozymes are a class of nanomaterials that mimic and achieve the function of natural enzymes (Figure 1B) (Zhou et al., 2017). Nanozyme-based water treatment methods have many advantages over protein enzymes (Tian et al., 2020). Nanozymes are able to operate both at high and low pollutant concentrations, which reduces sludge generation. In addition, nanozymes can work catalytically to a wide range of pollutants with low energy inputs. Although protein enzyme has many advantages, it should be pointed out that it also has some challenges, such as the high catalyst cost, low reusability, and tendence to deactivation. Compared with protein enzymes, nanozymes are attractive for both applied and fundamental research. A nanomaterial based on guanosine monophosphate coordinated copper mimics the activity of laccase and converts a diverse range of phenol-containing substrates as laccase, including catechol, hydroquinone, epinephrine, and naphthol (Liang et al., 2017). While the cost of this nanomaterial is about 2400-fold lower than that of laccase, its stability is overwhelming. Normally, these laccase mimics are nanozymes formed by copper and biological molecules, such as guanosine monophosphate, dipeptide, guanine-rich ssDNA, and proteins (Wang et al., 2019; Rashtbari and Dehghan, 2021; Tran et al., 2021; Xu et al., 2021). In the last two years, inorganic nanozymes appeared in the form of Cu-base metal-organic framework (MOF) (Shams et al., 2019; Hu et al., 2021) or CuO nanorods (Alizadeh et al., 2020) for efficient dye detection and phenolic pollutant degradation. Apparently, inorganic nanozymes are more robust than the ones involving biomolecules. They are abiotic and much stable at high salt, high temperature, and extreme pH and could be stored for a very long time.

### Enzyme-Mimicking POMs and POMs in Water Treatment

**FIGURE 2:**
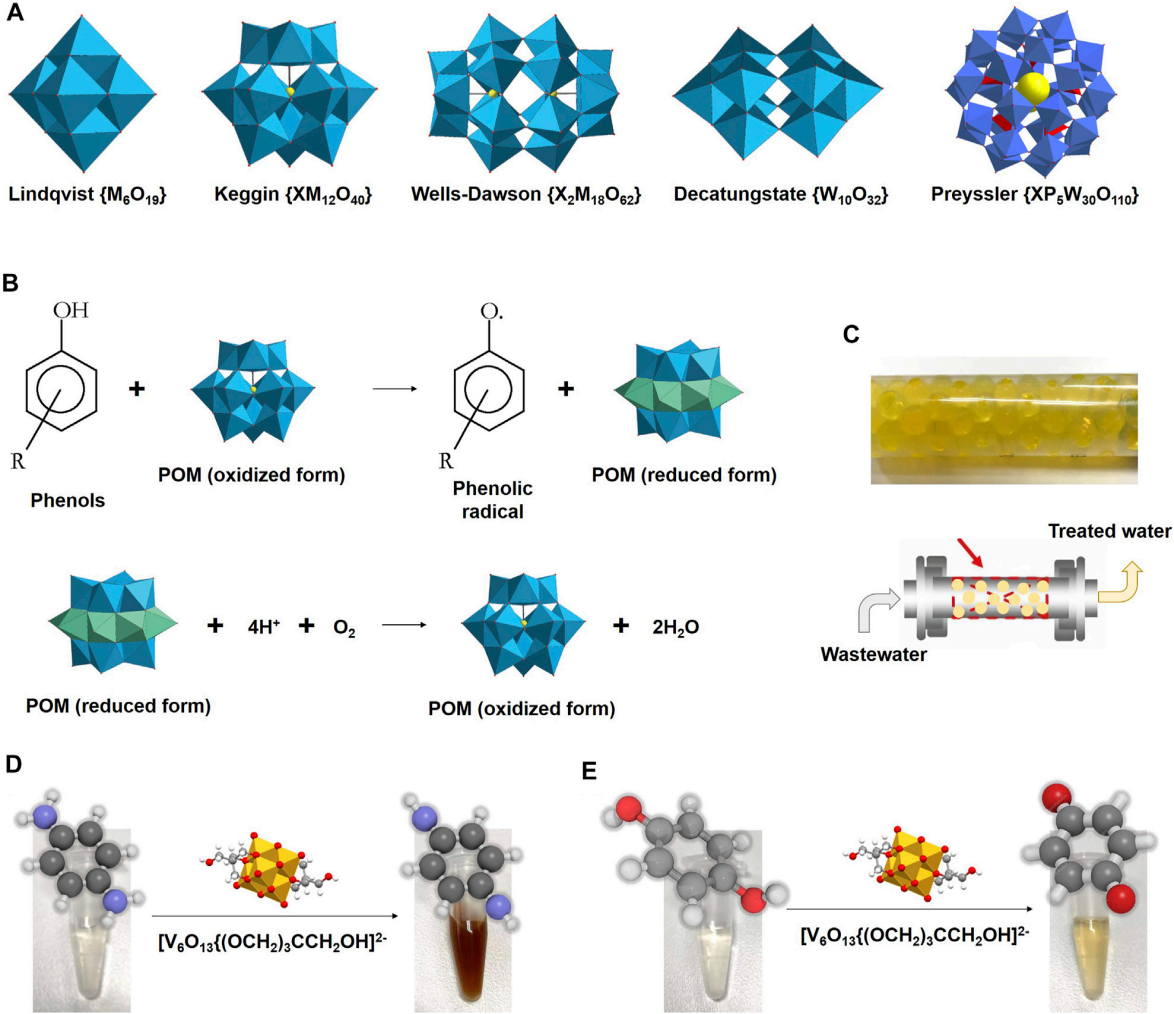
**(A)** Conventional types of polyoxometalates (M = Mo, W, V, Nb; X = heteroatom). **(B)** Scheme of degradation of phenolic contaminants by laccase-mimicking POMs. **(C)** Scheme of a batch reactor with POV hybrid as a quasi-homogeneous catalyst for degradation of emergent water contaminants. Scheme of laccase-like catalytic activity of POV hybrids for oxidating *p*-phenylenediamine **(D)** and hydroquinone **(E)**.

Polyoxometalates (POMs) are a kind of inorganic molecular materials with well-defined structures and mono-dispersity (Figure 2A). These nano-scaled clusters are composed of up to hundreds of face-, edge-, and/or angular shared bonds of metal-oxo polyhedral units. Most POMs can undergo reversible multi-electron redox reactions without structural change, which is a fantastic property in catalysis. Structures of POMs are rich and varying in morphisms and are versatile for co-assembly with other building blocks to construct diverse catalytic platforms. Some of them have nanopore or nanochannel structures with strong and/or selective affinity for guest molecules and/or ions, which makes them advantageous for size-selective catalysis. At the same time, POM surfaces are rich in oxygen, hydroxyl, and/or water ligands, suggesting that POM-based materials are excellent candidates for biomimetic applications. Over the last decades, POMs have demonstrated their promising biological activities, such as antitumoral (Bijelic et al., 2019), antimicrobial (Xu et al., 2020; Chen et al., 2021; Chen et al., 2022), insulin-sensitizing (Chen et al., 2020), immune-enhancing activities (Li et al., 2021; Li et al., 2022). In virtue of their structural diversity and physicochemical properties, POM-based nanomaterials exert oxidoreductase-mimicking activities, including oxidase, peroxidase (Chen K. et al., 2018; Jia et al., 2019), and catalase (Yadav and Singh, 2021). Compared with other nanomaterials, POMs are advantageous in their well-defined composition and molecular structure, as well as their economical production costs and mass production.

In addition, POM-based nanomaterials have been successfully applied in wastewater treatment. An electrocatalyst was prepared with polyoxometalate (POM) as a molecular platform on a large scale by a one-step pyrolysis method and showed its prospects in directly usage in seawater (Ma et al., 2017). A series of Ln/Cu-POMs were used for wastewater treatment with rapid adsorption and excellent selective separation of cationic dyes from aqueous solutions (Yi et al., 2015). Efficient heterogeneous photocatalytic materials, nanosized and bimodal porous polyoxotungstate-anatase TiO_2_ composites, were prepared and exhibited visible-light photocatalytic activities in degrading organophosphorus pesticides in aqueous solution (Li et al., 2005). POM-based hybrid materials showed great potential for the removal of contaminants, such as phthalates and bisphenol A (Figure 2B), from wastewater (Zou et al., 2021; Huo et al., 2022). These POM-based hybrid materials showed high stability and long duration in either continuous or separated modes for effective water remediation (Galiano et al., 2021; Lai et al., 2021). In addition, a voltametric sensor was prepared with the aid of POMs for the determination of simazine in wastewater samples (Ertan et al., 2016). Such hybrid materials combined with POMs offer new insights for designing functional materials with low cost and high efficiency for wastewater treatment.

### Challenges and Perspective

In recent years, POM has played a salient role in catalysis and showed its successful application in industrial catalysis, especially in the catalytic degradation of emerging contaminants in wastewater treatment. Structural diversity and excellent redox properties make POMs a vast treasure trove of active catalysts with intrinsic enzyme mimicking activities. The recent development of POM-based nanomaterials has created a new way for the development of artificial enzymes with high catalytic activity. Yet, people need to realize that nanozymes, including POMs, have not reached the catalytic activity as high as natural enzymes. There are several scientific and technologic challenges faced by POMs in catalytic degradation of endocrine disruptors. Since catalytic reactions involving nanozymes take place on the surface of the nanomaterials, surface modification represents an effective way to improve nanozyme activity. POMs are highly tailorable. Specifically, the capacity for POMs will be enlarged by the covalent or non-covalent interaction of POMs with a limitless range of organic moieties (Jia et al., 2017; Jia et al., 2018). POM organic derivatives have been shown to be able to assemble into a variety of hierarchical nanostructures applicable to different needs while maintaining their catalytic properties (Chen K. et al., 2018). Companied with low activity, the lack of reaction-specificity is another concern related to nanozymes, including POMs. POMs are able to catalyze the oxidation of a wide range of substrates and act as enzymes. However, the catalytic reactions involving nanozyme systems are typically more complex than natural enzymes. Laccase catalyzes substrate oxidation coupled to the four-electron reduction of molecular oxygen to water without releasing these partially reduced O_2_ products ROS. While the oxidation catalyzed by POMs may be coupled to the oxygen reduction to produce superoxides, •O_2_^−^ anions (Lai et al., 2021), in the real and complicated water environment. POMs are suitable for facile post-functionalization with other organic or inorganic molecules, which is an effective approach to design advanced catalytic materials. With rationale functionalization, POMs are expected to gain novel and improved physicochemical properties relevant to the development of novel catalysts for wastewater treatment in the near future.

## Discussion

Polyoxovanadates (POVs) are a subclass of POMs and have been described as bioinorganic drugs (Aureliano et al., 2021). POVs have shown different bioactivities not observed for monovanadate alone. Hybrid-type hexavanadate is one of the earliest organometallic POV derivatives that has been attracting research attention since its isolation. POV derivatives [V_6_^V^O_13_{(OCH_2_)_3_CCH_2_OH}_2_]^2−^ are redox stable and have been applied in homogeneous catalysis, materials science, and energy storage (Anyushin et al., 2020). Hybrid-type hexavanadates could be obtained through a simple, nontoxic, and one-pot method and showed favorable enzyme-like catalytic activity for oxidating phenylenediamine and hydroquinone (Figure 2). This oxidation could be conducted in a batch reactor with POV hybrid as a quasi-homogeneous catalyst (Figure 2C). [V_6_^V^O_13_{(OCH_2_)_3_CCH_2_OH}_2_]^2−^ is a versatile platform that can undergo DMAP-catalyzed esterification reactions with acid anhydrides to generate functional hybrid materials in catalysis. Following the etherate method for preparation and separation, solution stable POV hybrids were obtained and showed intrinsic laccase-like activities for catalyzing the oxidation of laccase substrate endocrine-disrupting *p*-phenylenediamine and hydroquinone to produce typical color changes (Figures 2D,E). These features POM-based hybrid catalysis as a potentially cost-effective approach for degradation of emergent water contaminants.

## Data Availability

The original contributions presented in the study are included in the article, further inquiries can be directed to the corresponding author.
